# Deletion at the *GCNT2* Locus Causes Autosomal Recessive Congenital Cataracts

**DOI:** 10.1371/journal.pone.0167562

**Published:** 2016-12-09

**Authors:** Bushra Irum, Shahid Y. Khan, Muhammad Ali, Muhammad Daud, Firoz Kabir, Bushra Rauf, Fareeha Fatima, Hira Iqbal, Arif O. Khan, Saif Al Obaisi, Muhammad Asif Naeem, Idrees A. Nasir, Shaheen N. Khan, Tayyab Husnain, Sheikh Riazuddin, Javed Akram, Allen O. Eghrari, S. Amer Riazuddin

**Affiliations:** 1 The Wilmer Eye Institute, Johns Hopkins University School of Medicine, Baltimore, MD, United States of America; 2 National Centre of Excellence in Molecular Biology, University of the Punjab, Lahore, Pakistan; 3 King Khaled Eye Specialist Hospital, Riyadh, Saudi Arabia; 4 Allama Iqbal Medical College, University of Health Sciences, Lahore, Pakistan; 5 National Centre for Genetic Diseases, Shaheed Zulfiqar Ali Bhutto Medical University, Islamabad, Pakistan; 6 McKusick-Nathans Institute of Genetic Medicine, Johns Hopkins University School of Medicine, Baltimore, MD, United States of America; University of Washington, UNITED STATES

## Abstract

**Purpose:**

The aim of this study is to identify the molecular basis of autosomal recessive congenital cataracts (arCC) in a large consanguineous pedigree.

**Methods:**

All participating individuals underwent a detailed ophthalmic examination. Each patient’s medical history, particularly of cataracts and other ocular abnormalities, was compiled from available medical records and interviews with family elders. Blood samples were donated by all participating family members and used to extract genomic DNA. Genetic analysis was performed to rule out linkage to known arCC loci and genes. Whole-exome sequencing libraries were prepared and paired-end sequenced. A large deletion was found that segregated with arCC in the family, and chromosome walking was conducted to estimate the proximal and distal boundaries of the deletion mutation.

**Results:**

Exclusion and linkage analysis suggested linkage to a region of chromosome 6p24 harboring *GCNT2* (glucosaminyl (N-acetyl) transferase 2) with a two-point logarithm of odds score of 5.78. PCR amplifications of the coding exons of *GCNT2* failed in individuals with arCC, and whole-exome data analysis revealed a large deletion on chromosome 6p in the region harboring *GCNT2*. Chromosomal walking using multiple primer pairs delineated the extent of the deletion to approximately 190 kb. Interestingly, a failure to amplify a junctional fragment of the deletion break strongly suggests an insertion in addition to the large deletion.

**Conclusion:**

Here, we report a novel insertion/deletion mutation at the *GCNT2* locus that is responsible for congenital cataracts in a large consanguineous family.

## Introduction

The ocular lens focuses light that passes through it onto the retina, where it is detected by photoreceptors and transformed into visual signals [1]. Cataract is an opacity or cloudiness caused by damage to the precise cellular structure of the lens, the precision of which is crucial to maintaining the transparency of the lens [2,3]. Light-blocking opacities compromise the function of the lens and reduce vision, often severely, unless the cataractous lens is removed [2,3]. Congenital cataracts are the primary cause of childhood blindness [2]. They occur in 1–6 cases per 10,000 live births; however, the incidence is much higher (5–15 cases per 10,000 live births) in developing countries [3,4].

Inherited cataracts comprise a significant fraction of the global burden of cataractogenesis [5]. Congenital cataracts may occur as an isolated anomaly only affecting the ocular lens or in conjunction with a developmental dystrophy of the anterior segment, such as microphthalmia or Peters anomaly [6]. Additionally, congenital cataracts have been associated with genetic multisystem disorders, such as Lowe syndrome and Nance-Horan syndrome [6]. More than 40 loci linked to human congenital cataracts have been mapped on different chromosomes [5]. Even phenotypically identical cataracts can be caused by mutations at different genetic loci (http://cat-map.wustl.edu/) and may exhibit different inheritance patterns [7].

Autosomal recessive congenital cataracts (arCC) have been associated with loci and genes on several chromosomes (1p, 1q, 3p, 3q, 6p, 7q, 8p, 9q, 11q, 16q, 17q, 19q, 20p, 21q, and 22q) [8–24]. Pathogenic mutations have been indentified in EPH receptor A2 (*EPHA2*), connexin50 (*GJA8*), FYVE and coiled-coil domain containing 1 (*FYCO1*), glucosaminyl (N-acetyl) transferase 2 (*GCNT2*), acylglycerol kinase (*AGK*), tudor domain containing 7 (*TDRD7*), crystallin alpha B (*CRYAB*), heat-shock transcription factor 4 (*HSF4*), galactokinase 1 (*GALK1*), lens intrinsic membrane protein 2 (*LIM2*), beaded filament structural protein 1 (*BFSP1*), crystallin alpha A (*CRYAA*), lanosterol synthase (*LSS*), crystallin beta B1 (*CRYBB1*), and crystallin beta B3 (*CRYBB3*) [9,10,13,17–19,21–29].

*GCNT2*, the gene implicated in this study, encodes the I-branching enzyme, a beta-1,6-N-acetylglucosaminyltransferase that converts fetal i antigen in erythrocytes to the adult I antigen [13]. The i antigen is a linear poly-N-acetyllactosamine chain, whereas the I-antigen structure contains branched poly-N-acetyllactosaminoglycans [30,31]. First discovered in human red blood cells with cold-agglutinating autoantibodies [32,33], the I and i antigens are carbohydrate structures attached to glycolipids and glycoproteins on the cell surfaces of various tissues and body fluids [31,34]. Normally red blood cells predominantly express the I antigen on the cellular surface; however, in patients with the adult i phenotype, red blood cells express low levels of I antigen and high levels of the i antigen [35].

Yamaguchi and colleagues were among the first to report the association of the adult i phenotype with congenital cataracts [36]. To date, multiple mutations in *GCNT2* have been identified in patients with congenital cataracts. These include both missense and nonsense mutations in *GCNT2* [35,37–39]. Moreover, deletions of coding exons of *GCNT2* have been reported [35,40]. Here, we report the causal mutation in a large, consanguineous familial case of congenital cataracts. With linkage analysis, we localized the disease interval to a region on chromosome 6p harboring *GCNT2*, and failure to amplify the gene in affected individuals suggested an insertion/deletion at the locus. Whole-exome sequencing coupled with chromosome walking confirmed a deletion of approximately 190 kb, including *GCNT2*.

## Materials and Methods

### Patient Recruitment and Clinical Evaluation

Consanguineous Pakistani families with cataracts were recruited to participate in a collaborative study to understand the genetic aspects of arCC. Institutional Review Board approval was granted by the National Eye Institute (Bethesda, MD), the Johns Hopkins University School of Medicine (Baltimore, MD), and the National Centre of Excellence in Molecular Biology (Lahore, Pakistan). All participating subjects gave informed written consent consistent with the tenets of the Declaration of Helsinki.

A detailed medical history was compiled for each participant by interviewing family members and reviewing available medical records. Ophthalmic examinations, including slit-lamp microscopy, were performed at the Layton Rahmatulla Benevolent Trust Hospital in Lahore, Pakistan. All participating members donated approximately 10 ml of blood, drawn into 50 ml Sterilin Falcon tubes with 20 mM EDTA. Genomic DNA was extracted as previously described [41,42].

### Exclusion Analysis

All documented arCC loci and genes were excluded by genotyping with 70 polymorphic short tandem repeat markers. Amplification reactions for the exclusion analysis was performed as described previously [41,42]. The amplified products from each DNA sample were mixed with a loading cocktail containing 400HD size standards (Applied Biosystems, Foster City, CA), and resolved on an ABI PRISM 3100 Genetic Analyzer (Applied Biosystems). Genotypes were assigned with GeneMapper software (Applied Biosystems).

### Linkage Analysis

Two-point linkage analysis was performed using the FASTLINK version of MLINK from the LINKAGE Program Package (provided in the public domain by the Human Genome Mapping Project Resources Centre, Cambridge, UK) [43,44]. Maximum logarithm of odds (LOD) scores were calculated using PLINK (Shaun Purcell, Boston, MA). arCC was analyzed as a fully penetrant trait with an affected allele frequency of 0.001. The marker order and distances between the markers were calculated from the chromosome 6 sequence maps published by the NCBI (National Center for Biotechnology Information, Bethesda, MD).

### Sanger Sequencing for Mutation Screening

Primer pairs for individual exons were designed using the Primer3 program. The primer sequences and amplification conditions are available upon request. The PCR amplifications were performed in 10 μl reactions with 20 ng genomic DNA as described previously [41,42]. The PCR primers for each exon were used for bidirectional sequencing with the BigDye Terminator Ready Reaction Mix, in accordance with the manufacturer’s instructions (Thermo Fisher Scientific, Waltham, MA).

Sequencing products were dissolved in 10 μl of Formamide (Applied Biosystems) and resolved on an ABI PRISM 3100 Genetic Analyzer (Applied Biosystems). Sequencing results were assembled with ABI PRISM sequencing analysis software, version 3.7, and analyzed with SeqScape software (Applied Biosystems).

### Chromosome Walking

A total of 23 primer pairs spanning the *GCNT2* locus were designed using Primer3 software (Table 1). PCR amplifications were performed in 10 μl reactions with 20 ng genomic DNA, 1 μl of 10 μM of forward and reverse primers, 1 μl of 10x PCR buffer (100 mM Tris HCl (pH 8.4), 400 mM NaCl, 15 mM MgCl_2_, and 2.5 mM spermidine), 250 μM dNTP mix, 700 mM dimethyl sulfoxide (DMSO), 500 mM betaine, and 0.2 U Taq DNA polymerase. PCR amplification consisted of a denaturation step at 95°C for 5 minutes followed by a two-step touchdown procedure. The first step of 10 cycles consisted of denaturation at 95°C for 30 s, followed by primer-set-specific annealing for 30 s, and elongation at 72°C for 45 s. The annealing temperature (provided in Table 1) decreased by 1°C per cycle. The second step of 30 cycles consisted of denaturation at 95°C for 30 s, followed by annealing (10°C below annealing temperature used in the first step) for 30 s and elongation at 72°C for 45 s. The last step was a final elongation at 72°C for 5 minutes. The PCR products were separated on a 1.5% agarose gel and visualized on a UV transilluminator to determine the amplification.

**Table 1:** Primer sequences for the confirmation of *GCNT2* deletion breakpoints in PKCC215.

Primer Pair	Direction	Sequence	Annealing Temperature (°C)	Region	Product Size (bp)
a	F	GCCTCTGCGAAAGTGAAATG	68	10413447–10413854	407
	R	TTGCAGCTGAGAATGTTAGGC			
b	F	TCCCCTTCTATTAGCCTGCTC	68	10462475–10462795	320
	R	GGCTCTTCTGCCATGTAAGG			
c	F	GGAAATCATCCTTGCAGAGC	68	10465833–10466212	379
	R	GAGGTGCCTTTGTGTTTGTG			
d	F	TGGCTTGGGTTCTATTCTGG	68	10466314–10466622	308
	R	TCCAGAGCTGTGAGCGAATA			
e	F	TCAACCCTGACACAGTGCTT	68	10467543–10467908	365
	R	GCACATGTCACTCTGCGTTA			
f	F	GGAACCGTGAGCCAATTAAA	70	10468290–10468674	384
	R	GCAGCAGCTAACTGGTCTCC			
g	F	AGACCAGTTAGCTGCTGCAC	68	10468657–10468929	272
	R	TGCCCTGCTAATTTTTGTATTTT			
h	F	GATTACTACCAGGGCTGTGGAC	70	10468697–10468879	184
	R	GCCAGACTGGTCTTGAACTTCT			
i	F	CAGGGCTGTGGACTGTTGTA	70	10468706–10469140	434
	R	CTGAGCAGGGGACACAACTA			
j	F	ACATGGCTGCCTGTCTTCA	70	10469216–10469578	362
	R	CACCATCTGTGGTAGGCAGA			
k	F	ACCACATCATCTGCTAAAACG	70	10472212–10472581	369
	R	TTGTTTTGGGTAGTGTTGACAT			
l	F	GTGCCAGCTGTGTGACTGTT	70	10488488–10488871	383
	R	CCCAGGAGACATTCAGTGCT			
m	F	TGCATTGCGTTAGCTGAATC	70	10501483–10501879	396
	R	ACACCAGGGTTCAGGTCCTA			
n	F	AGAGGTCTGGGCAATCTCTG	70	10653054–10653438	384
	R	GTTTCCCTTGGTGGCAACT			
o	F	GCACTGGGAACTTGGAAATG	70	10654655–10655048	393
	R	TGGCTCTTCCATGGACTGTT			
p	F	AAATGCTGCCTGGATGTTG	70	10656747–10657142	395
	R	TCCCAAACTGCGATCTATGA			
q	F	GTGTGCCCACATCTCTTCCT	68	10657266–10657693	427
	R	GGTTGGATTTGGACCATGAG			
r	F	CAGAGCAGGACTCCATCTCAAT	68	10657573–10657768	195
	R	GTAGAAGCAGCCAAAAAGGAAA			
s	F	AACCTGCCACCTGTTCTTGT	68	10657690–10658111	421
	R	CCCTAGCAGTGATCCCATGT			
t	F	GATCACTGCTAGGGCTGGAG	68	10658098–10658490	392
	R	GTTTCCCTCTGCACCAGAAA			
u	F	TTTCTGGTGCAGAGGGAAAC	68	10658471–10658887	416
	R	CACAGGACAAGCTCTGAAAACA			
v	F	TGTCTACTGGGGCCTGTCA	70	10659000–10659449	449
	R	CACCACGTCCGGCTACTTT			
w	F	CCACATCCACCCTGGAATTA	68	10663284–10663680	396
	R	ATGGGGTTGGAAGCCATTAT			

**Note:** F: forward; R: reverse.

### Whole-Exome Library Preparation and Sequencing

Whole-exome sequencing libraries were prepared using the Nextera Rapid Capture Expanded Exome kit in accordance with the manufacturer’s protocol (Illumina, San Diego, CA). Genomic DNA was quantified with a Fluorometer (Qubit 2.0; Invitrogen, Carlsbad, CA). The enriched libraries were quantitated with an Agilent 2100 Bioanalyzer (Palo Alto, CA) and pooled together in equal concentrations. The pooled, bar-coded exome libraries were clustered using the TruSeq v3 Cluster Kit at a 13 pM concentration, and paired-end sequenced (2 × 100 bp) using the TruSeq SBS Kit v3 on a HiSeq 2000 sequencing system (Illumina).

### Sequence Alignment and Variant Calling

Bioinformatic analysis of exome data was performed with Lasergene Genomics Suite (DNASTAR, Madison, WI). The raw reads were mapped to the human genome (GRCh38) using DNASTAR’s proprietary assembly software (SeqMan NGen 12) with default parameters. The mapped reads were subjected to ArrayStar, to filter the variants based on genotype, sequencing depth, conservation, and read-quality parameters. Additional filtering steps were applied to remove any variants with a minor allele frequency greater than 0.1% in public databases, including dbSNP, 1000 Genomes, exome variant servers (NHLBI server and ExAC browser), or in our in-house exome datasets of ethnically matched control samples and individuals from unrelated familial cases without cataracts.

### Real-time Expression Analysis

The use of mice in this study was approved by the Johns Hopkins Animal Care and Use Committee (ACUC), and all experiments were performed by a protocol approved by the Johns Hopkins ACUC. Mouse lenses were obtained at different developmental stages, including embryonic day 15 (E15), day 18 (E18), at birth (P0), postnatal day 3 (P3), day 6 (P6), day 9 (P9), day 12 (P12), day 14 (P14), day 21 (P21), day 28 (P28), day 42 (P42), and day 56 (P56). Mouse lenses were obtained as described previously [41,42]. Total RNA from the lenses was isolated with TRIzol (Invitrogen) according to the manufacturer’s instructions. The quality and quantity of the total RNA were determined on a NanoDrop Lite Spectrophotometer (Thermo Fisher Scientific). First-strand cDNA synthesis was completed with the SuperScript III First-Strand Synthesis Kit according to the manufacturer’s instructions (Thermo Fisher Scientific). Quantitative real-time PCR was performed on a StepOne Real-Time PCR System using predesigned *Gcnt2* TaqMan expression assays (Applied Biosystems). *Gapdh* served as an endogenous internal control. The 2^-ΔCT^ method was used to determine the relative expression of *Gcnt2*, normalized to *Gapdh* expression, at each developmental stage.

## Results

A large consanguineous family (designated PKCC215 in our cohort), which includes six individuals who have congenital cataracts and nine unaffected individuals, was recruited from the Punjab province of Pakistan. Medical records and interviews with the family indicated that all affected individuals developed cataracts during infancy (Table 2). Ophthalmic examinations of the participating members of PKCC215, conducted with slit-lamp microscopy, detected bilateral nuclear cataracts in every affected individual.

**Table 2:** Clinical characteristics of individuals of PKCC215 who exhibit autosomal recessive congenital cataracts.

Individual ID	Gender	Age of onset	Age at enrollment	Visual Acuity (OD/OS)	Clinical Findings
12	F	birth	3 months	PL/PL	B/L nuclear cataracts
15	M	birth	5 years	CF/CF	B/L nuclear cataracts; B/L nystagmus
16	F	birth	8 months	CF/PL	B/L cataracts

**Note:** Age of onset determined by the age at which the first symptoms manifested; OD: oculus dexter; OS: oculus sinister; PL: perception of light; CF: counting fingers; B/L: bilateral.

**Fig 1:**
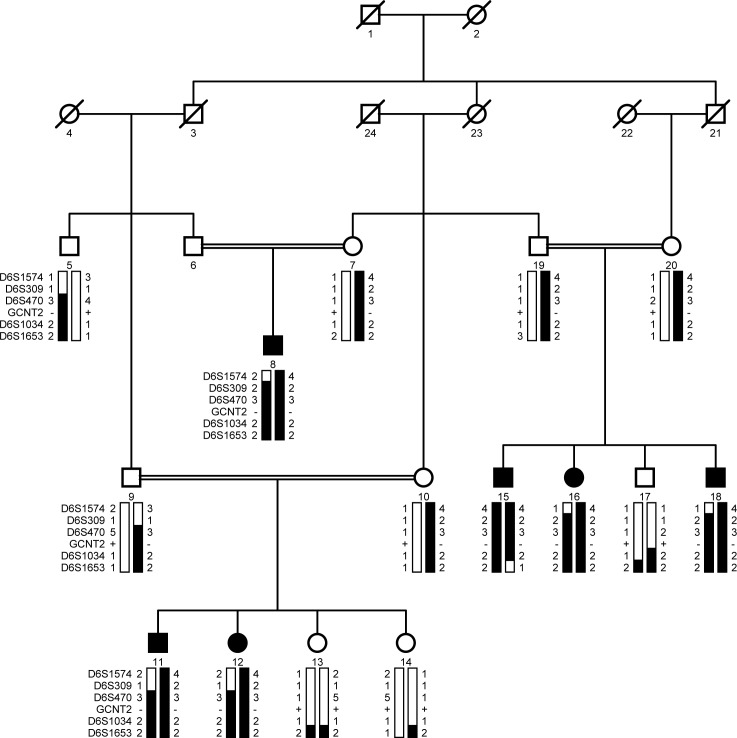
Pedigree diagram of the family, PKCC215. The haplotypes of 5 adjacent chromosome 6p microsatellite markers are shown. Alleles constituting the risk haplotype are shaded black, and those not segregating with cataracts are shown in white. Square: male; circle: female; filled symbol: affected; double line between symbols: consanguineous mating; diagonal line through symbol: deceased.

Initially, we screened PKCC215 for linkage to all loci and genes reported to be associated with arCC with fluorescently labeled short tandem repeat (STR) markers. Homozygosity-based analysis of the resultant alleles suggested linkage to chromosome 6p markers. Maximum two-point LOD scores of 5.78 and 5.53, at the recombinant fraction θ = 0, were obtained with the markers D6S470 and D6S309, respectively (Table 3). Haplotypes of each family member were constructed with the alleles of the STR markers, which supported the results of linkage analysis, localizing the cataractous phenotype to an interval of 12.64 cM (6.22 Mb) on chromosome 6p, between D6S309 and D6S1653 (Fig 1). Every affected individual was homozygous for the D6S470 and D6S1034 markers, while unaffected individuals were either heterozygous or homozygous for the wild-type sequence (Fig 1).

**Table 3:** Two-point LOD scores of PKCC215 and microsatellite markers used for linkage analysis.

Marker	cM	Mb	0	0.01	0.05	0.09	0.1	0.2	0.3	Z_max_	θ_max_
D6S1574	9.18	6.01	**−∞**	0.89	1.86	1.95	1.94	1.50	0.91	1.95	0.09
D6S309	14.07	8.22	5.53	5.43	5.00	4.55	4.43	3.23	1.94	5.53	0.00
D6S470	18.22	10.02	5.78	5.69	5.22	4.75	4.63	3.34	2.03	5.78	0.00
D6S1034	23.23	12.2	**−∞**	3.41	3.64	3.41	3.33	2.38	1.34	3.64	0.05
D6S1653	26.71	14.44	**−∞**	-2.91	-0.90	-0.26	-0.16	0.33	0.34	0.34	0.30

The localization interval on chromosome 6p harbors *GCNT2*, a gene previously associated with cataractogenesis as well as with the adult i phenotype. The sequencing of all five exons of *GCNT2*, the exon–intron boundaries, and the 5′ and 3′ UTR regions were completed for the unaffected members of PKCC215; however, PCR reactions with the genomic DNA of all six affected individuals failed to yield specific PCR products. It is notable that the multiplex PCR reactions of the six affected individuals did generate specific products for every primer set except that of *GCNT2* (data not shown).

To further investigate the possibility of a deletion mutation at the *GCNT2* locus in PKCC215, we examined the whole-exome data of three affected individuals, which revealed two absent variants on the chromosome 6p interval. The two absent SNPs, rs35318586 (chr6: 104,650,35 bp) and rs3756954 (chr6: 107,245,60 bp), identified the proximal and distal boundaries of a deletion.

**Fig 2:**
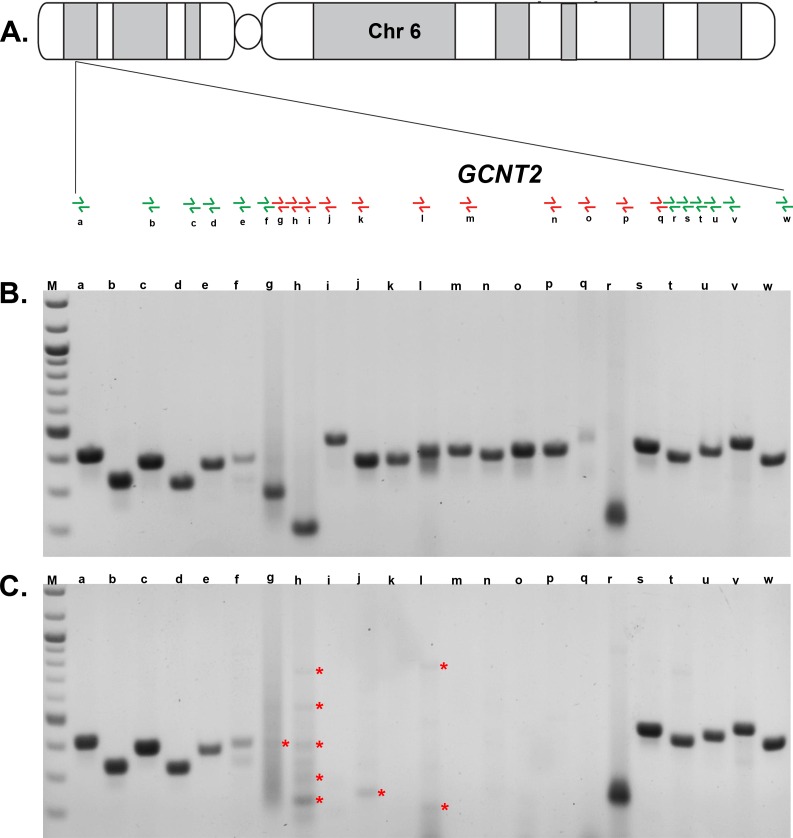
Characterization of the *GCNT2* deletion responsible for congenital cataracts in PKCC215. **A**) Model of chromosome 6 indicating the placement of the primer pairs in the PCR amplification of the *GCNT2* region. Visualization (on a 1.5% agarose gel) of the amplification products from each primer pair with the DNA of **B**) an unaffected and **C**) affected member of PKCC215. Amplification of the genomic DNA of the affected individual was successful (produced specific PCR products) with the primer pairs indicated in green. Amplification of the affected individual’s DNA failed (non-specific or no PCR products) with the primer pairs indicated in red. Note: Asterisk indicates non-specific PCR products.

To further confirm the deletion and refine the proximal and distal boundaries, we designed 23 primer pairs spanning *GCNT2* and the flanking 5′ and 3′ regions (Fig 2A and Table 1). The primer pairs were used to amplify the genomic DNA of an unaffected parent, individual 7, and an affected child, individual 8. As shown in Fig 2, the PCR reactions distal to primer pair “f” and proximal to primer pair “r” failed to amplify the genomic DNA of the affected individual. Subsequently, we tested the genomic DNA of the other five affected individuals (11, 12, 15, 16, and 18) with the same 23 primer pairs. The results of the PCR reactions with the DNA of these five individuals were equivalent to those of affected individual 8; amplification reactions with primer pairs annealing to genomic DNA on chromosome 6 in the 10468657–10657693 region failed to yield specific products (data not shown). The causal deletion (10468657–10657693) on chromosome 6p harbors *GCNT2*, *RP1-290I10*.*5* (long intergenic non-coding RNA), *RP11-360O19*.*4* (antisense non-coding RNA), and *RP11-360O19*.*5* (a processed pseudogene). Among the deleted species, only *GCNT2* is expressed in the ocular lens, based on both the next-generation transcriptome sequencing [45,46] and proteome profiling of the developing mouse lens (unpublished data).

To identify the proximal and distal breakpoints of the deletion, we set out to amplify across the deletion with the forward primer from the “f” primer pair and the reverse primer from the “r” primer pair (the new combination was designated “LR1”). We designed an additional primer pair, “LR2”, to amplify across the deletion with a forward primer annealing proximal to the forward primer of LR1 and a reverse primer annealing distal to the reverse primer of LR1. We attempted to amplify the genomic DNA of each of the six affected individuals with both the LR1 and LR2 primer pairs. None of the 12 PCR reactions, with primer pairs LR1 and LR2 or their reciprocal combinations, yielded specific products. The failure to amplify across the deletion indicates that the affected members of PKCC215 carry a complex chromosomal rearrangement at the *GCNT2* locus comprised of a large deletion accompanied, most likely, by an insertion.

**Fig 3:**
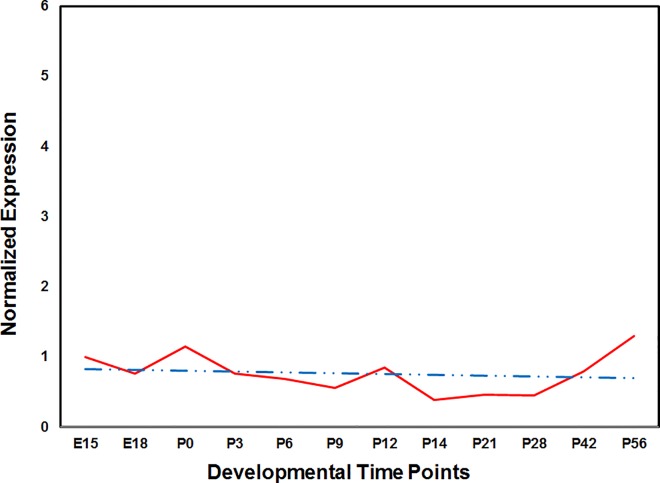
Expression profile of glucosaminyl (N-acetyl) transferase 2 in the developing mouse lens. The expression of *Gcnt2* at different developmental time points was normalized to *Gapdh*. The x-axis and y-axis represent the developmental time point and the normalized expression level of *Gcnt2* mRNA, respectively. Note: red line: expression of *Gcnt2*; blue dashed line: linear regression.

As a first step in understanding the physiological significance of *GCNT2* in the development of the ocular lens, we explored the expression of *Gcnt2* in our recently published mouse lens transcriptome. *Gcnt2* is expressed as early as embryonic day 15, and low but consistent expression was present at all six time points examined in the study [45]. To further investigate the expression profile of *Gcnt2*, we performed quantitative real-time PCR analysis at six additional postnatal time points, using the expression level at E15 as a reference for the comparison of the subsequent 11 time points. As shown in Fig 3, expression levels of *Gcnt2* remain steady throughout the early developmental period. Such consistent expression suggests a critical role for *GCNT2* in the development of the lens and the maintenance of its transparency.

## Discussion

Here, we report a novel deletion in *GCNT2* that causes autosomal recessive congenital cataracts. Initial exclusion analysis localized the critical interval to chromosome 6p with statistically significant two-point LOD scores. Next, PCR amplification of the coding exons of *GCNT2* suggested a large deletion at the locus. The deletion was further confirmed by a lack of chromosome 6p variants in the exomes of affected individuals. Chromosome walking delineated the deletion to approximately 190 kb on chromosome 6p, encompassing *GCNT2*, which segregates with the cataractous phenotype in the family.

**Fig 4:**
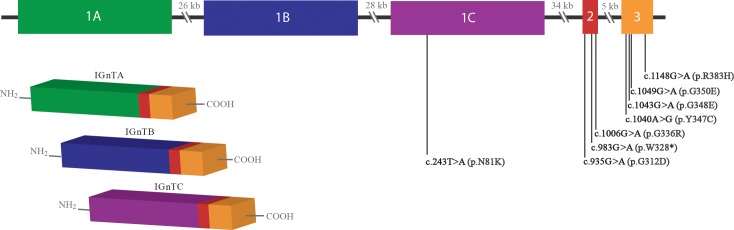
A schematic of the *GCNT2* isoforms generated through alternative splicing. *GCNT2* extends 135 kb on chromosome 6p24.3–24.2, and produces three isoforms through alternative splicing that differ in their first coding exon (1A, 1B, or 1C). To date, 10 different causal mutations have been identified in *GCNT2*, including seven missense mutations, one nonsense mutation, and two large genomic deletions (Table 4). Mutations residing in exon 2 or 3 generate both the adult i phenotype and congenital cataracts.

*GCNT2*, also named *IGnT*, extends 135 kb on chromosome 6p24.3–24.2, and alternative splicing produces three isoforms that each contain a different initial coding exon (1A, 1B, or 1C), followed by exons 2 and 3, which are common to the three transcripts [35,47]. To date, 10 different causal mutations have been identified in *GCNT2*, including seven missense mutations, one nonsense mutation, and two large genomic deletions (Fig 4 and Table 4). Interestingly, mutations residing in the first exon result in the adult i phenotype without congenital cataracts whereas the mutations in exons 2 and 3 result in both the adult i phenotype and congenital cataracts. Yu and colleagues recently proposed a partial association of the two traits, the adult i phenotype, and congenital cataracts. They suggested that a defect in only the C isoform, IGnTC, leads to the reduction of I antigen in red blood cells, whereas a deficiency in the function of all three IGnT enzymes results in cataractogenesis [48].

**Table 4:** Mutations reported in *GCNT2*.

No.	Nucleotide Change	Amino Acid Change	Coding Exon	Phenotype	Reference
1	c.243T>A	p.N81K	1C	adult i	[50]
2	c.935G>A	p.G312D	2	arCC; adult i	[38]
3	c.983G>A	p.W328*	2	arCC; adult i	[13]
4	c.1006G>A	p.G336R	2	cataract; adult i	[37]
5	c.1040A>G	p.Y347C	3	arCC	[39]
6	c.1043G>A	p.G348E	3	arCC; adult i	[35]
7	c.1049G>A	p.G350E	3	cataract; adult i	[37]
8	c.1148G>A	p.R383H	3	arCC; adult i	[35]
9	deletion	-	1B, 1C, 2, 3	arCC; adult i	[35]
10	deletion	-	1B, 1C, 2, 3	arCC; adult i	[40]

**Note:** arCC: autosomal recessive congenital cataracts.

In 2001, Yu and colleagues published three Taiwanese pedigrees with the adult i phenotype; each member of the three families who exhibited the adult i phenotype also manifested congenital cataracts [35]. The expression of the I antigen in blood is regulated only by the IGnTC isoform of *GCNT2*. Therefore, mutations in exon 1A or 1B have not been associated with the adult i phenotype [35,47]. In their report, Yu and colleagues suggested that the association between the adult i phenotype and congenital cataracts may be due to a functional dependency of I-related and cataract-related genes, or might be a pleiotropic effect of the adult i phenotype gene on cataractogenesis [48].

Chen and colleagues developed a mouse model lacking the third exon of *Gcnt2* to investigate the physiological function of the enzyme [49]. However, the *Gcnt2* knockout mice did not develop cataracts during early development and, although older mice manifested mild cataracts in the cortical regions, there was no difference in the degree of opacity between the lenses of wild-type and knockout mice [49]. Nevertheless, the genetic data associating cataractogenesis with mutations in *GCNT2* remains strong. The discrepancy between the cataractous phenotypes between patients with *GCNT2* mutations and that of the *Gcnt2*-deficient mice might be due to tolerance or compensation for *GCNT2*-encoded glycosyltransferases. Li and colleagues suggested that differences in the function or expression of other glycosyltransferases in mice might be responsible for the lack of cataractogenesis in the *Gcnt2* knockout [48].

In conclusion, we have identified a large deletion at the *GCNT2* locus spanning ~190 kb that is responsible for autosomal recessive congenital cataracts in a large family. This is the largest deletion reported at the *GCNT2* locus and is likely accompanied by an insertion. Identifying the proteins, such as GCNT2, that are essential for the maintenance of lens transparency is vital for understanding and preventing cataractogenesis. The identification of new genes associated with cataractogenesis will help us to better understand the biology of the ocular lens at a molecular level.

## Supporting Information

S1 File The ARRIVE checklist has been completed for the manuscript. (PDF)
