# Correction: Deletion at the *GCNT2* Locus Causes Autosomal Recessive Congenital Cataracts

**DOI:** 10.1371/journal.pone.0173719

**Published:** 2017-03-09

**Authors:** Bushra Irum, Shahid Y. Khan, Muhammad Ali, Muhammad Daud, Firoz Kabir, Bushra Rauf, Fareeha Fatima, Hira Iqbal, Arif O. Khan, Saif Al Obaisi, Muhammad Asif Naeem, Idrees A. Nasir, Shaheen N. Khan, Tayyab Husnain, Sheikh Riazuddin, Javed Akram, Allen O. Eghrari, S. Amer Riazuddin

There are errors in the fourth paragraph of the Results section. The correct paragraph is: To further investigate the possibility of a deletion mutation at the *GCNT2* locus in PKCC215, we examined the whole-exome data of three affected individuals, which revealed the absence of variants at chromosome 6 interval harboring *GCNT2*. A search of variants in proximal and distal regions of *GCNT2* identified two SNP’s rs35318586 (chr6: 10,464,803bp) and rs3756954 (chr6: 10,724,327bp) in exome data defining the proximal and distal boundaries of the deletion.
